# Highly diversified expansions shaped the evolution of membrane bound proteins in metazoans

**DOI:** 10.1038/s41598-017-11543-z

**Published:** 2017-09-28

**Authors:** Misty M. Attwood, Arunkumar Krishnan, Markus Sällman Almén, Helgi B. Schiöth

**Affiliations:** Department of Neuroscience, Functional Pharmacology, Uppsala University, BMC, Box 593, 751 24 Uppsala, Sweden

## Abstract

The dramatic increase in membrane proteome complexity is arguably one of the most pivotal evolutionary events that underpins the origin of multicellular animals. However, the origin of a significant number of membrane families involved in metazoan development has not been clarified. In this study, we have manually curated the membrane proteomes of 22 metazoan and 2 unicellular holozoan species. We identify 123,014 membrane proteins in these 24 eukaryotic species and classify 86% of the dataset. We determine 604 functional clusters that are present from the last holozoan common ancestor (LHCA) through many metazoan species. Intriguingly, we show that more than 70% of the metazoan membrane protein families have a premetazoan origin. The data show that enzymes are more highly represented in the LHCA and expand less than threefold throughout metazoan species; in contrast to receptors that are relatively few in the LHCA but expand nearly eight fold within metazoans. Expansions related to cell adhesion, communication, immune defence, and developmental processes are shown in conjunction with emerging biological systems, such as neuronal development, cytoskeleton organization, and the adaptive immune response. This study defines the possible LHCA membrane proteome and describes the fundamental functional clusters that underlie metazoan diversity and innovation.

## Introduction

Integral transmembrane proteins are essential components involved in various biological processes such as signal transduction, transport of various solutes, enzymatic activity, cell-cell recognition, and cell attachment. Indeed, they are theorized to be particularly important in the evolution of metazoans and the complex developments of cell signalling, stable cell adhesion and cell communication^1^. Furthermore, membrane proteins have been involved in the advancement of complexity in systems such as immune response with TOLL-like receptors and the immunoglobin superfamily members^2^ as well as the nervous system with proteins such as ligand-gated ion channels^3^. Membrane proteins also play significant roles in disease, including cancers and metabolic diseases, and as such are heavily targeted by pharmaceutical drugs. For example, membrane receptors alone make up 44% of the human drug targets^4^.

Previously, comparisons of metazoan genomes with unicellular representatives in the context of crucial membrane protein families has revealed that a vast number of these genomic features are unique to animals, while a substantial proportion also has a pre-metazoan origin. For example, receptor tyrosine kinases, essential for maintenance of the multicellular condition through cell-cell communication, has undergone dramatic expansions in unicellular holozoans prior to the onset of metazoan multicellularity^5,6^. The G protein-coupled receptors (GPCR), however, have undergone rapid expansions close to the origin of metazoans while only a minimal number of representatives were identified in unicellular holozoans and other opisthokonts such as Fungi^7,8^. Other expansions of specific membrane protein families projected to be involved in metazoan evolution have also been identified such as extracellular matrix proteins^9^ among others. However, while individual families and superfamilies of membrane proteins have been studied in detail, there are still families that have not been studied at all or the studies have been limited to a specific set of species, in particular vertebrates. Hence, to our knowledge there does not exist a current resource that provides a comprehensive overview of the evolution of the human membrane proteome and compares this throughout metazoans and unicellular holozoans. Such large scale comparisons of membrane proteomes across animals and its closest unicellular relatives will contribute a global view on the evolution and diversification of membrane proteomes. This includes: (1) the families that are truly metazoan innovations; (2) families that have undergone lineage specific expansions; and (3) in particular, the families that are conserved throughout all analysed taxa thus constituting the functional hub of the membrane proteome. In addition, such an approach will allow us to reconstruct the membrane proteome component potentially present in the last holozoan common ancestor (LHCA) before the emergence of metazoans.

In this study, we extend our previous human proteome characterization^10^ to classify membrane proteins to an additional 21 metazoan species to include 5 vertebrate and 17 invertebrate species, and additionally the two closest unicellular representatives from the opisthokont lineage to understand how the membrane bound proteome evolved during multicellular transition and metazoan morphological complexity. Using a two-step clustering process and manual curation, we classified the membrane proteomes and annotated the functional clusters. As such, we have mapped the evolution and diversification of the membrane proteome, and have potentially reconstructed the membrane proteome component present in the LHCA and traced its evolution in various lineages. Further, we define 604 conserved functional clusters that are predominantly found in all analysed taxa, constituting the conserved component of the membrane proteome. Overall, we present a comprehensive comparative analysis of the membrane proteomes in holozoa and delineate the major trends in membrane proteome evolution that are concomitant with the evolution of metazoan morphological complexity. This comprehensive dataset, which features species specific innovations as well as the conserved complement, will facilitate further exploration and advance our understanding of the role of the membrane proteome in metazoan multicellularity.

## Results

We have investigated 24 eukaryotic proteomes that in total comprise 499,841 proteins, of which 123,014 (25%) are predicted to be membrane proteins. The average number of membrane proteins of the 24 species is 4965 proteins (Table 1: Membrane Proteome Totals). The relative size of the membrane proteome varies between 33% for *M*. *musculus* and 17% for *A*. *pisum* and correlates with the size of the whole proteome (rho = 0.85, p < 2.17 e −06, Spearman correlation).

**Table 1:** Membrane Proteome Totals.

Species	Total Proteins	Total Membrane proteins	% Membrane proteins	LHCA subset	Metazoan innovation	Species specific innovation
*H sapiens*	20,894	5,797	28	4,129	1,668	53
*M musculus*	22,627	7,359	33	4,731	2,628	170
*G gallus*	16,354	4,461	29	3,511	950	111
*X tropicalis*	18,442	5,773	31	4,345	1,428	34
*D rerio*	25,638	7,865	31	6,054	1,811	233
*C intestinalis*	16,671	3,542	21	2,408	1,134	649
*S purpuratus*	28,842	7,437	26	6,082	990	0
*D melanogaster*	13,918	3,658	26	2,653	1,005	381
*A mellifera*	15,314	2,926	19	2,215	711	1
*A pisum*	35,189	5,867	17	3,492	2,375	990
*D pulex*	30,137	5,243	17	3,281	1,962	1172
*C elegans*	20,447	6,788	33	3,297	3,491	1720
*C teleta*	32,175	7,968	24	5,713	2,255	910
*L anatina*	34,105	8,881	26	5,891	2,990	1120
*C gigas*	26,092	6,081	23	4,507	1,574	714
*L gigantea*	23,675	5,378	23	3,806	1,572	536
*I linei*	8,720	1,588	18	1,328	260	255
*S mansoni*	10,772	2,576	24	1,829	747	465
*N vectensis*	24,773	5,568	22	4,496	1,072	379
*T adhaerens*	11,520	3,146	27	2,602	544	244
*M leidyi*	16,548	4,219	25	3,064	1,155	680
*A queenslandica*	29,883	5,861	20	4,279	1,582	760
*M brevicollis*	9,196	2,854	31	2,108	—	502
*C owczarzaki*	8,758	2,543	29	2,013	—	408

Columns include: total number of proteins, total number predicted membrane proteins, percent of membrane proteins, protein totals of LHCA subset per species, as well as metazoan innovations and species specific innovations protein totals from the full dataset (includes No Description and DUF clusters).

### Clustering

**Figure 1:**
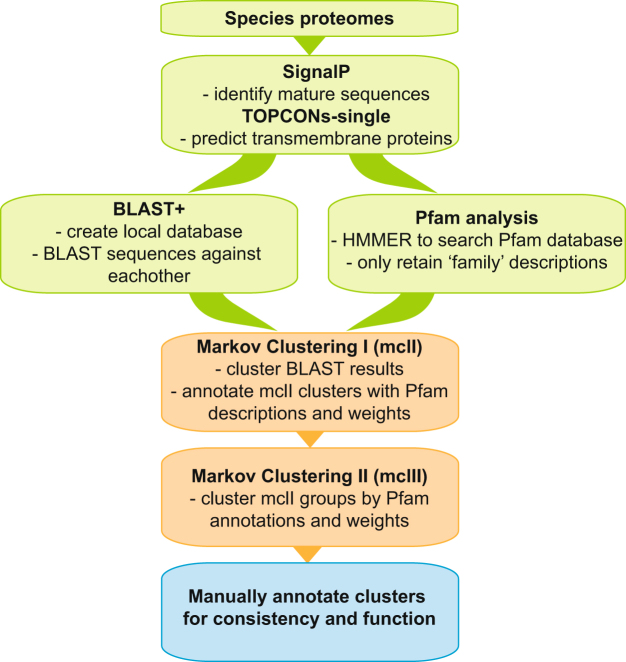
Methods pipeline for dataset. Pipeline of methods used to create dataset for analysis. The 24 species proteomes were downloaded and prepared for analysis. SignalP was used to excise signal peptides and TOPCONs-single consensus software predicted the transmembrane proteins. An all-versus-all BLAST database was created as well Pfam annotations were searched for all sequences. Two-step Markov clustering was performed on first the BLAST results and then on those clusters using the Pfam annotation. The first and second clusters were manually annotated for consistency and function using our human membrane proteome characterizations as well as Pfam descriptions.

To identify potential families of evolutionarily related membrane families we first conducted clustering using the MCL algorithm to build groups based on sequence similarity obtained from BLAST searches (see Fig. 1: Methods pipeline for dataset). This first level of sequence similarity clustering resulted in 115,104 membrane proteins being sorted into 6609 homologous groups plus 7910 proteins into singlet clusters. Out of all membrane proteins, 50% are found in a first level cluster with 50 or more sequences (total 357 clusters). The largest first level cluster contains 2172 proteins and is described as vertebrate olfactory receptors.

To detect more distant relationships, we used the MCL algorithm again to build groups of clusters from the first level of sequence similarity clustering that were based on shared Pfam families. In total, the second level clustering and characterization resulted in 3083 described clusters based on detailed functional descriptions from the human protein homolog in combination with Pfam family annotation or based solely on inference from human protein characterizations. Hence after first and second level clustering, 105,757 membrane proteins were functionally annotated into 2181 groups that ranged from 1 to 8911 members.

### Functional Classification

**Figure 2:**
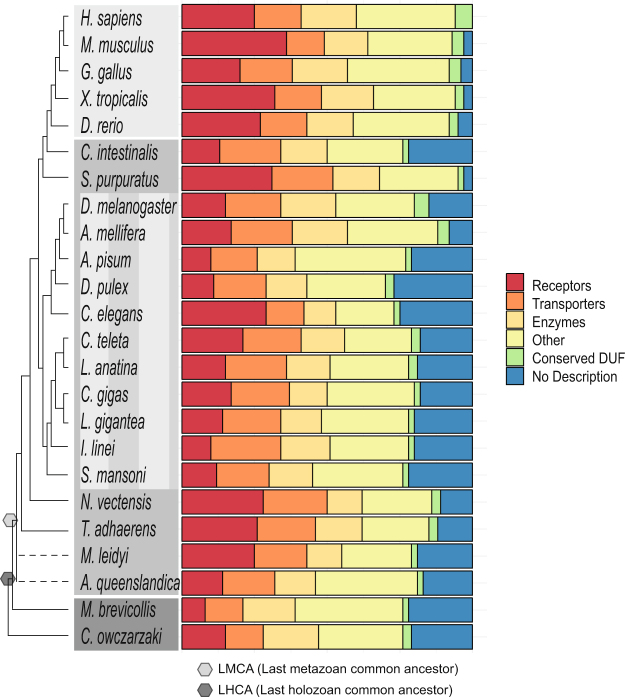
Functional Classifications Overview. The functional classification groups are depicted as a bar chart relative to the total size of each species membrane proteome. The last metazoan common ancestor (LMCA) (light gray pentagon) and last holozoan common ancestor (darker gray pentagon) are noted. The dendrogram represents the evolutionary relationship among the investigated organisms. The branch lengths are not relative to evolutionary distance. The dashed lines indicate unresolved lineages. Species topology including *I*. *linei* is based on ref.^60^ and the *A*. *queenslandica* placement is from ref.^61^. The light gray colour box corresponds to vertebrate metazoans, the darker gray represents invertebrate species, the striped gray box is protostomia members, and the dark gray are the two opisthokont unicellular relatives.

The membrane protein dataset and the identified clusters were classified through several factors: the detailed characterization of the human membrane proteome (see Methods for details) and Pfam family annotations. The human membrane proteome was first characterized into one of the main functional classes of *receptors*, *enzymes*, *transporters*, or else into *other functional classes*. The human protein characterizations involved in each first level clustering were assessed and a consensus first level cluster description was obtained. The second level clusters were then controlled for conflicting classifications of the underlying proteins/clusters and an appropriate characterization for each second level cluster was determined. The coverage of the functional classes in each species can be found in Fig. 2: Functional Classifications Overview.

### Receptors

Of the 105,757 characterized proteins, the entire receptor class contains a total of 25,862 proteins and has the largest proportion of proteins (24%) in comparison with the other main functional classes; enzymes (17%) and transporters (21%). There are eleven species, including all five vertebrates, in which the receptor class consists of the most number of proteins in their membrane proteome, ranging from 20% to 36% of their classified proteins. In contrast, the two outgroup holozoan species, *M*. *brevicollis* and *C*. *owczarzaki*, contain much less with 8% and 15% respectively.

**Figure 3:**
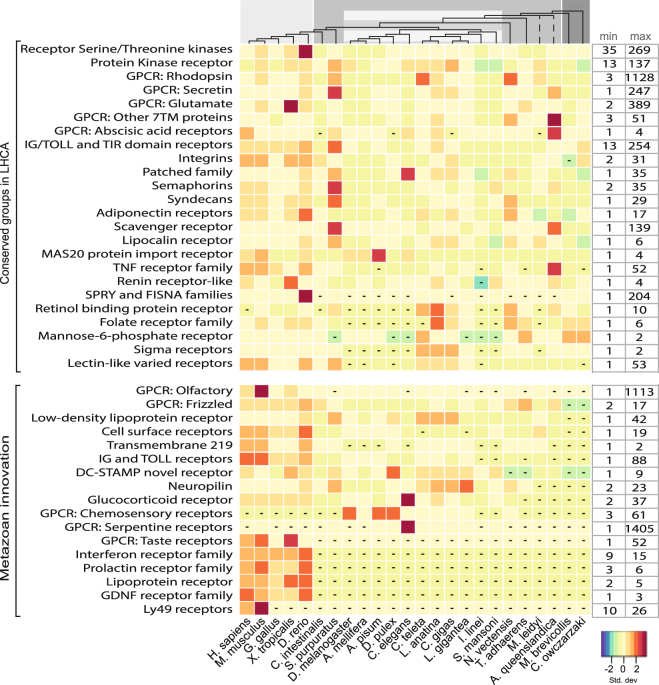
Receptors: the evolution of selected receptor families across eukaryotes. The heatmap illustrates the relative content of selected receptor families across species. For each organism, the number of receptors in a specific family was standardized with the standard deviation for that family. According to the key, an increasing red hue indicates more receptors coded as the number of standard deviations from zero (the mean) and an increasing blue colour corresponds with less receptor proteins coded. The boxes with dashes indicate zero members in that cluster. The receptor families are described on the left and functional clusters are grouped according to those identified in the LHCA subset and those clusters that are metazoan innovations. The minimum and maximum values for the selected receptor families are displayed on the right side of the heatmap so that the relative colour differentiation is clarified by the range of proteins present in each transporter family. The dendrogram at the top of the heatmap represents the evolutionary relationship among the investigated organisms. The branch lengths are not relative to evolutionary distance. The dashed lines indicate unresolved lineages. Species topology including *I*. *linei* is based on ref.^60^ and the *A*. *queenslandica* placement is from ref.^61^. The light gray colour corresponds to vertebrate metazoans, the medium gray represents invertebrate species, the striped gray box indicates protostomia species, and the dark gray are the two opisthokont unicellular relatives.

The GPCR superfamily is the largest receptor group with 16,153 identified proteins that are further divided into families (see Fig. 3: Receptors). The origin of the GPCRs is ancient and the breadth of this superfamily is evident in the multitude of proteins in the 22 metazoan species, which aside from 56 members in the extremely small sized membrane proteome of *I. linei*, ranges from 132 to 1919 identified sequences per species. This is in contrast to *M*. *brevicollis* and *C*. *owczarzaki* that have 17 and 58 sequences identified, respectively. Another large group is the receptor kinases with 3385 proteins, which includes the receptor serine/threonine kinases and protein kinase receptors such as the receptor tyrosine kinases.

### Transporters

**Figure 4:**
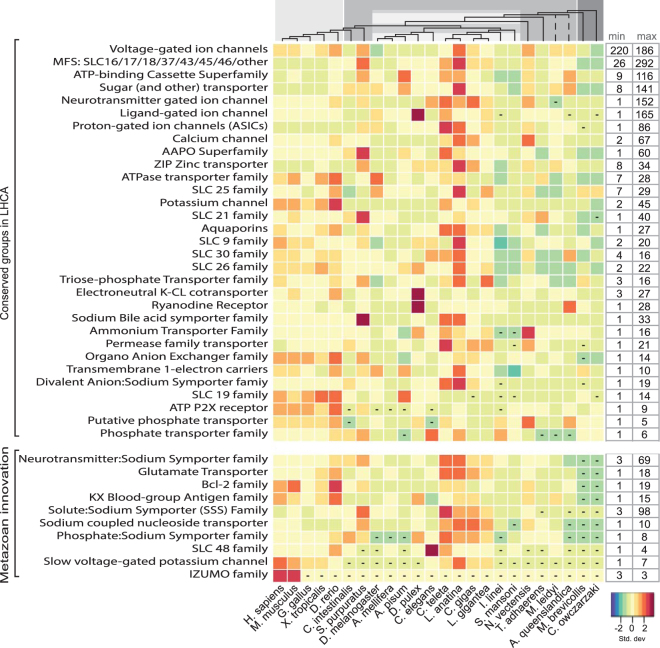
Transporters: the evolution of selected transportation families in eukaryotes. The heatmap presents the relative content of selected transporter families across different metazoan species. For each organism, the number of transporters in a specific family was standardized with the standard deviation for that family. The key indicates that an increasing darker red hue illustrates more transporters coded as the number of standard deviations from zero (the mean) and an increasing blue colour corresponds with less transporter proteins coded. The boxes with dashes indicate no members in that group. The transporter family names are specified on the left side left and functional clusters are grouped according to those identified in the LHCA subset and those clusters that are metazoan innovation. The minimum and maximum values for the selected transporter families are displayed on the right side of the heatmap so that the relative colour differentiation is clarified by the range of proteins present in each transporter family. The dendrogram at the top of the heatmap represents the evolutionary relationship among the investigated organisms. The branch lengths are not relative to evolutionary distance. The dashed lines indicate unresolved lineages. Species topology including *I*. *linei* is based on ref.^60^ and the *A*. *queenslandica* placement is from ref.^61^. The light gray colour corresponds to vertebrate metazoans, the darker gray represents invertebrate species, the striped gray box is protostomia members, and the dark gray are the two opisthokont unicellular relatives.

The transporters are the second largest functional class with 22,362 sequences, constituting 21% of the total classified proteins. The transporters are the largest functional class for eleven of the invertebrate metazoan species, ranging from 16% to 21% of their classified membrane proteins. The selected transporter families shown in Fig. 4: Transporters have been divided into groups such as active transporters, channels, solute carriers (SLCs) with further subdivisions, porters and unknown biochemical mechanism. Solute carrier families such as the Major Facilitator Superfamily contribute a major portion of the transporters and show significant patterns of expansions in the lophotrochozoa species. For brevity we have condensed several of the SLC groups, incorporating some of the SLC families into larger subgroups based on the descriptions major facilitator super (MFS) family and the amino acid permeases (APC).

### Enzymes

**Figure 5:**
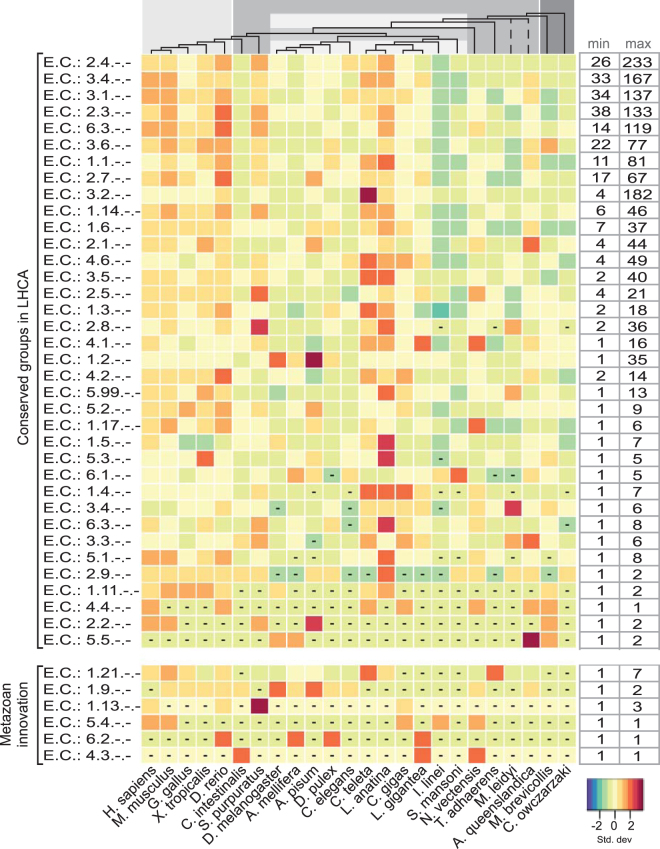
Enzymes: the evolution of the human enzyme repertoire across eukaryotes. The heatmap illustrates the relative content of transmembrane enzymes throughout different species. For each organism, the number of enzymes in each family was standardized with the standard deviation for that family. The key illustrates that an increasing darker red hue indicates more enzymes coded as the number of standard deviations from zero (the mean) and an increasing blue colour corresponds with less enzyme proteins coded. The boxes with dashes signify no species members were found in the group. The Enzyme Commission (EC) classes are described on the left side. They are grouped according to those identified in the LHCA subset and those clusters that are metazoan innovations. The minimum and maximum values for the selected enzyme families are displayed on the right side of the heatmap so that the relative colour differentiation is clarified through the range of proteins present in each enzyme family. The dendrogram at the top of the heatmap represents the evolutionary relationship among the investigated organisms. The branch lengths are not relative to evolutionary distance. The dashed lines indicate unresolved lineages. Species topology including *I*. *linei* is based on ref.^60^ and the *A*. *queenslandica* placement is from ref.^61^. The light gray colour corresponds to vertebrate metazoans, the darker gray represents invertebrate species, the striped box indicates protostomia species, and the dark gray are the two opisthokont unicellular relatives.

Although the majority of enzymes are soluble, the membrane proteomes hold a considerable number of proteins from this class with 18,827 proteins in total (18%) (See Fig. 5: Enzymes). The enzymes are classified according to the Enzyme Commission (EC) system, which is a hierarchical system that classifies proteins according to the type of reaction they catalyse and their substrate. The EC system has four levels of which we present the first two, which generally only considers the type of catalytic reaction. Out of the six top level EC classes, the transferases (EC 2.-) are the most abundant with 6885 proteins identified. Glycotransferases (EC 2.4) are the largest subgroup with 2976 proteins and acyltransferases (EC 2.3) are substantial with 1896 identified. Hydrolases (EC 3.-) are another sizeable group with 6814 proteins and has several large subgroups, including esterases (EC 3.1) with 1973 proteins and proteases (EC 3.4) with 2405 proteins. The isomerases (EC 5.-) contain the least number of enzymes with 354 identified proteins in total.

### Other functional characterizations

**Figure 6:**
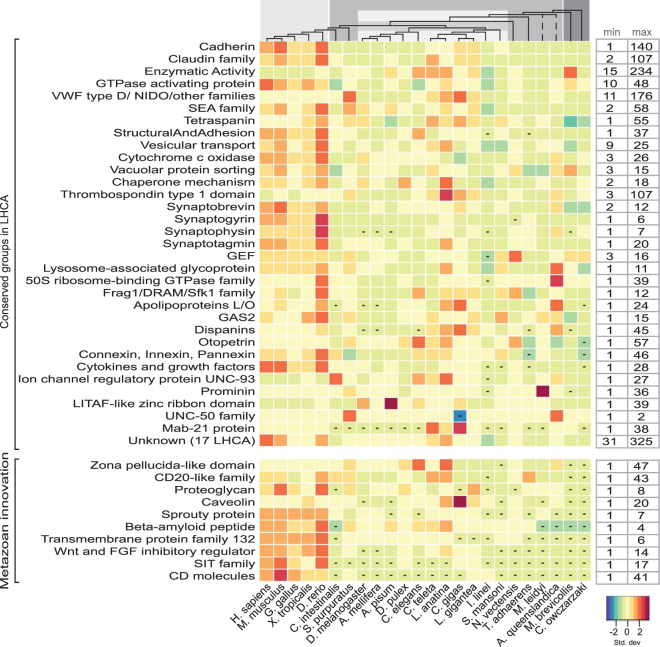
Other functional clusters: the evolution of protein families classified by various functions. The heatmap illustrates the relative content of protein families with functions other than receptors, transporters, or enzymes. For each organism, the number of proteins in a certain family was normalized with the standard deviation for that family. The key indicates an increasing darker red hue as more receptors coded as the number of standard deviations from zero (the mean) and an increasing blue colour corresponds with less proteins. The dashes in boxes indicate that are no species members for that cluster. The family names are indicated on the left side of the heatmap and functional clusters are grouped according to those identified in the LHCA subset and those clusters that are metazoan innovations. The minimum and maximum values for the selected families are displayed on the right side of the heatmap so that the relative colour differentiation is clarified through the range of proteins present in each family. The dendrogram at the top of the heatmap represents the evolutionary relationship among the investigated organisms. The branch lengths are not relative to evolutionary distance. The dashed lines indicate unresolved lineages. Species topology including *I*. *linei* is based on ref.^60^ and the *A*. *queenslandica* placement is from ref.^61^. The light gray colour corresponds to vertebrate metazoans, the darker gray represents invertebrate species, the striped gray box indicate protostomia species, and the dark gray are the two opisthokont unicellular relatives.

There are 35,485 proteins that could not be categorized into the *receptors*, *transporters*, or *enzymes* classes, but still had an associated function and were subsequently categorized within the *other functional* class. Included in this group are Annotated Singlet functional clusters which include 452 proteins that belong to species specific clusters. Additionally, there are 3571 proteins identified with an unknown but conserved Pfam protein or domain family, also known as a Domain of Unknown Function (DUF). Specific groups of proteins are highlighted in Fig. 6: Other functional clusters while further descriptions of the Annotated Species Specific Singlet clusters are described in Supplemental Dataset S1 and the Conserved DUF proteins are shown in Supplemental Table S2. One interesting functional cluster contains several groups that total 1969 proteins identified with having miscellaneous enzyme functions, where these proteins are not formally categorized with an EC number, but they are described as primarily having some enzymatic functional activity. *M*. *brevicollis* has a rather large group of 234 proteins identified in this category, where 162 proteins belong in one cluster which also has thirteen other species with identified proteins (maximum of 37 proteins) in this cluster. Several of the Pfam annotations include proteins found in humans; however this cluster did not include any human homologues during the BLAST clustering. The Pfam descriptions include families such as RNA ligase (PF09414), Poly (ADP-ribose) polymerase catalytic domain (PF00644), TATA-element modulatory factor DNA and TATA binding (PF12329 and PF12325), and RNase_T exonuclease family (PF00929) among others. These conserved protein families could be involved in the *brevicollis* RNA repair system^11^, in RNA editing^12^, or even possibly employed in RNA-mediated genome rearrangements^13^.

### Comparative analysis of membrane protein families

The expansions of membrane protein families in different organisms and lineages were studied in a comparative perspective. To determine the functional clusters that describe membrane proteins which are strongly suspected to have existed in the last holozoan common ancestor (LHCA), we analysed clusters in which at least one protein was identified in either unicellular holozoan opisthokont outgroup and also present in metazoan species. These conserved functional groups comprise 604 clusters in total that describe 90,906 membrane proteins across all the investigated species. This indicates that at least 74% of the predicted membrane proteins are described in these clusters that potentially extend to the origin of holozoans.

### The membrane proteome of the last common holozoan ancestor

The membrane proteome of the last common holozoan ancestor was evaluated by averaging the number of proteins (rounding up) in the 604 functional clusters in *M*. *brevicollis* and *C*. *owczarzaki* (see Supplemental Data S1 for details). These 604 conserved functional clusters describe 2283 proteins we presume to comprise the fundamental core of the membrane proteome of the last common ancestor of holozoans. It should be noted that the proposed LHCA proteome will not include proteins that have been lost from the LHCA, and thus we identify proteins that are likely to be present but may not comprise all of it. Aside from the *other functional classification* group that contains a variety of different types of functional clusters, the *enzymes* are the largest classified group in the LHCA membrane proteome with 36 functional clusters describing 517 proteins, which is 23% of the fundamental core of the LHCA membrane proteome. Transferases (171 proteins) and hydrolases (191 proteins) appear to be the most prolific enzyme groups, with peptidases (EC 3.4; 65 proteins), esterases (EC 3.1; 54 proteins), and glycosyltransferases (EC 2.4; 63 proteins) being the most abundant. However, all six broad EC classifications are present in the LHCA membrane proteome. *Transporters* are the second largest group that comprises 370 proteins from 95 different clusters, which is 16% of LHCA membrane proteome. The active transporters include the largest single cluster with the ATP-binding cassette superfamily comprising 39 proteins. Solute carrier proteins that are within the Major Facilitator Superfamily also show a strong presence with 26 proteins in one combined cluster. The *receptor* class is the smallest with 309 proteins but they are divided among 31 clusters and compose just 14% of the membrane receptome. There are several large clusters that dominate the receptor class: the IG/TOLL and TIR domain receptors with 125 proteins and Protein kinase receptors and Receptor Serine/Threonine kinases with 33 and 58 proteins, respectively. The GPCR superfamilies present in the last common ancestor include Glutamate, Rhodopsin, Secretin, Abscisic acid and Other 7TM proteins and total 38 proteins.

The *other functional classification* group has 1088 proteins among 398 different clusters and includes activities such as GTPase activating proteins, SEA family, Thrombospondin type 1 domain, Cadherins, Tetraspanins, and Vesicular transport among others. One sizable combined group (31 proteins) is described by Pfam annotations such as Rab-GTPase-TBC domains and GTP-ase activator protein for Ras-like GTPase, which may be involved as activating proteins of Rab-like small GTPases. And 75 proteins are identified with a conserved Pfam DUF domain with unknown functional activity.

### Metazoan Expansion

From the 31 clusters identified in the *receptor* class in the LHCA, many of the receptor family proteins in metazoan lineages doubled or expanded more than eight times in comparison to the LHCA with 309 receptor proteins. GPCR superfamilies such as Rhodopsin, Glutamate, and Secretin show significant specific species expansions, such as in *S*. *purpuratus*, *C*. *teleta*, *and N*. *vectensis* with more than 1000 proteins each. Additionally, the lophotrochozoans show distinct increases in particularly rhodopsins but also secretin GPCRs. Serine/Threonine kinase receptors also show expansions, particularly in the vertebrate species, while protein receptor kinases exhibit more pronounced expansions and possible losses throughout the metazoan lineages. The *transporter* clusters doubled or more than tripled in numbers throughout metazoan evolution in comparison to the 369 transporters identified in LHCA. Many families show varied expansions and possible losses throughout metazoans. For example, Neurotransmitter gated ion channels showed increases within lophotrochozoans (ranging from 91 to 152 proteins) as well as several other species specific expansions in *N*. *vectensis* and *C*. *elegans*, 106 and 103, respectively. In comparison, vertebrates contain about 43 to 48 proteins, with *D*. *rerio* containing 71. In fact, there appears to be a pattern of expansions within the lophotrochozoans, a few specific increases in other species, and then a consistent range within the vertebrates throughout the transporter class. One exception to this pattern is acid-sensing proton-gated ion channels, which actually show a marked decrease in these proteins within vertebrates in comparison to invertebrates. In the metazoan lineages, *enzyme* classifications slightly more than doubled in comparison to the 517 enzymes described in the LHCA. Once again, as in the transporter class, there are general trends that show increases within the lophotrochozoans and several specific species expansions, with a consistent range within vertebrates. It can be seen that particular classifications which are the largest group in the LHCA proteome show pronounced increases across metazoans, including transferases (EC 2.3, EC 2.4, and EC 2.7) and hydrolases (EC 3.1, EC 3.2, and EC 3.4). There are two exceptions to the general pattern of increases in receptor and enzyme proteins throughout metazoans in comparison to the predicted LHCA proteome: *I*. *linei* and *S*. *mansoni*. The number of enzymes and receptors decreased in these species to 268 and 398, respectively. The predicted membrane proteome sizes are two of the smaller ones in the dataset with 1588 and 2576 proteins, so large expansions might not be expected, however *A*. *mellifera* also has only 2926 predicted membrane proteins and has expanded all three of the functional classes, i.e., enzymes, receptors, and transporters to 569, 498, and 600 proteins, respectively.

### The rise of metazoan specific membrane proteins

Metazoan specific *receptors* (i.e., novel receptor families not identified in the LHCA), which includes 5752 proteins found throughout the 22 metazoan species, increase in abundance the most throughout animal evolution in comparison to *transporter* (2045 proteins) and *enzyme* (75 proteins) innovative groups. As previously known, various GPCR superfamilies significantly expand throughout metazoans, for example the novel olfactory family expand in insecta and vertebrate families, reaching 1113 membrane proteins in *M*. *musculus* and 392 proteins in *X*. *tropicalis*. IG and TOLL receptors also expand significantly in vertebrates as well as interferon families and cell surface receptors. *Transporters* specific to metazoans include 2045 proteins, and as in many conserved LHCA transporter classes, here again significant expansions in the lophotrochozoan species as well as *S*. *purpuratus* can be seen. The Neurotransmitter:Sodium Symporter Family and the Solute:Sodium Symporter (SSS) Family shows variances throughout metazoan species (ranging from 3 to 36 members); however the Lophotrochozoans as well as *S*. *purpuratus* contain 36 to 98 members.

There are five specific *enzyme* EC classifications that are found solely in the metazoan lineages. These include three oxidoreductase classes: EC 1.13 (that act on oxygenases), EC 1.21 (that act on X-H and Y-H to form an X-Y bond), EC 1.9 (that act on a heme group of donors) plus carbon-nitrogen lyases (EC 4.3), intramolecular transferases (EC 5.4), and also acid-thiol ligases (EC 6.2). The *other* classification group includes 6264 proteins that are specific to metazoan lineages and found in at least two different species. These include many different functional activities from numerous clusters. There are expansions found throughout metazoans and examples include clusters identified with wnt signalling, the MAGE family, CD20-like family, Sprouty protein, immunoglobin families, the SIT family, and spermatogenesis among others. Figure 6: Other functional clusters presents several of the metazoan specific clusters and Supplemental Data S1 describes the full dataset.

### Species specific DUF, No Description, and Annotated Singlet functional clusters

There are 17,257 sequences that are classified Uncharacterized with No Description as no further information could be gleaned about them. There are several large singlet clusters, for example *C*. *elegans* has one unique cluster with 72 members, another cluster with 25 members, and two clusters that each contain 19 sequences. These large clusters may provide intriguing insights into novel protein family expansions within a species. 3571 sequences are identified in specific DUF clusters, with at least 127 clusters containing 10 or more members from the different species. Supplemental Table S2 presents the proteins annotated as Uncharacterized (No Description), as well as Conserved DUF functional clusters.

## Discussion

Here, we provide an extensive comparative analysis of the evolution of membrane proteomes in holozoans. We identified 604 functional clusters that are found conserved throughout metazoans by integrative analysis of 123,014 membrane proteins, and characterized 86% of the dataset (105,757 proteins). We propose that the last holozoan common ancestor (LHCA) contains at least 2200 conserved membrane protein components found throughout 604 functional clusters, while at least 1004 novel families have been formed during later metazoan evolution. With one exception in the simplified orthonectid *I*. *linei*, the membrane proteomes have increased throughout metazoan species, nearly doubling in size and even tripling in several species compared with the LHCA.

What can be clearly seen in this study are the patterns of expansions, losses, and conservations, which are highlighted in Figs 3, 4, 5 and 6. In the enzyme classes, both vertebrates and lophotrochozoans exhibit distinct increases, however in the transporter groups, the four lophotrochozoan species as well as *S*. *purpuratus* show many pronounced expansions throughout the clusters while vertebrates do not expand as significantly. And in the receptor classes, it is obvious that vertebrates have undergone greater expansions and innovations than the other represented species. Specifically in vertebrates we see large expansions related to cell-cell adhesion (cadherins, semaphorins, syndecans); communication (cytokines and growth factors, GTPase activating proteins, serine/threonine kinase receptors, GEF proteins); adaptive immune defence (TOLL and IG receptors, immunoglobin superfamily, Tumour necrosis factor family, CD molecules, CD20-like family, Lectin-like receptors); and developmental processes (proteins involved in spermatogenesis, wnt signalling, Strabismus proteins). A recent analysis suggests that the last metazoan common ancestor (LMCA) underwent vast increases in protein-protein interaction complexity that is mediated via membrane proteins^1^ and here we see that this is evident for protein families involved in protein-protein interactions such as cadherins, tetraspanins, and integrins that extensively expand throughout metazoans. Lophotrochozoans also show distinct expansions even greater than vertebrates in many clusters such as dispanins, tetraspanins, proteins with the Thrombospondin domain (TSP-1), as well as specific ion channels. The expansions in lophotrochozoans of TSP-1 correlates with a study that showed proteins with the TSP-1 domain aid in bivalve byssus extensibility^14^ as well as being involved in the evolution of hemolymph/blood concentrations^15^. Additionally the numerous expansions in ion channels are proposed to function in isosmotic regulatory responses and the mechanisms of pH regulation and calcification^16^.

Interestingly, 82% of the characterized membrane proteins are identified in one of the conserved functional clusters, which suggests that a substantial proportion of the diversity within the eukaryotic membrane proteome was already present in the LHCA. However, while many essential protein families are present before metazoan expansion and innovation, we see that there are major differences in how the enzyme, transporter, and receptor functional classes evolve. The LHCA proteome contains a relatively low percentage of receptors with 11% in comparison to enzymes comprising 19% and transporters with 14%. Conversely, metazoans show tremendous receptor expansions with 25% in humans and up to 36% in *M*. *musculus*, while enzyme proteins average only 15% and the transporters average 19%. The lophotrochozoan species, which show expansions in several transporter classes, still only average 20% transporters in their proteome.

The rapid development of the membrane receptome from just 8% in the choanaflagellate *M*. *brevicollis* and 15% in the filastereate *C*. *owczarzaki* species to significantly higher fractions in metazoans highlights the importance of membrane bound proteins in the evolution from unicellular to multicellular organisms. In particular, unique expansions in the receptomes of vertebrates can be seen in GPCR receptors including taste, olfactory, frizzled, the GDNF family, cell surface receptors, nuclear hormone receptors, and in the vertebrate IG and TOLL receptors (see Fig. 3: Receptors). The increasing receptor repertoire propelled several key developments including cell adhesion, immune response, and may have enabled the evolution of complex body plans and tissues by providing efficient and specific signalling between cells. For example, DC-STAMP receptors, which originated in metazoans, are involved in immune response and also in cell-cell fusion of osteoclasts^17^. The gain of multiple receptor families and their swift expansion in metazoan lineages suggests strong positive selection towards more diverse signalling systems during animal evolution^18^ and the novel vertebrate innovations evolutionary advantage of an adaptive immune response.

Interestingly, approximately 30% of the receptors in the predicted LHCA proteome are identified in protein kinase signalling, as tyrosine kinase signalling is an essential element of signal transduction and intercellular signalling in multicellular animals and which aided in the development of more complex systems^19^. Furthermore, a quite recent study in *C*. *owczarzaki*, which has three temporally distinct cell types, concluded that phosphorylation via Serine/Threonine and Tyrosine kinase networks facilitate unicellular temporal differentiation using post-translational gene regulation^20^. As the mechanisms that unicellular ancestors use to regulate protein abundance and the activity of different proteins according to type are still being investigated, the plethora of kinases in the receptor proteome of the last common ancestor is an important avenue to explore in regards to conserved phosphosignaling and proteome regulation.

Distinct innovations and expansions are shown in membrane proteins that correlate with the development of specific biological systems. Proteins involved in neuronal and brain development such as receptor serine/threonine kinases, neuregulins, and integrins seem to have first emerged in the unicellular holozoans and are found conserved across metazoan lineages and which we show further vertebrate enlargements and species specific novel innovations^21–23^. Indeed, the number of protein kinase receptors show significant increases specifically in lophotrochozoans, reaching up to four times as many as those found in the LHCA, while Receptor Serine/Threonine kinases expand more notably in vertebrates. This is consistent with the fact that the nervous system evolved early in the animal evolution and has been complemented by the addition and introduction of several neuronal gene families, including different receptors and voltage gated ion channels^24,25^. For example, we see the emergence and relatively consistent number of proteins across species identified in the novel metazoan myelin proteolipid protein (PF01275) cluster, which are involved in developing the central nervous system and myelination^26^. Plasmolipin, which is also involved in myelination, shows expansions solely in vertebrates with up to 6 proteins identified. Another novel metazoan cluster include proteins in the wnt/beta-catenin signalling pathways, which drives myelin gene expression and myelinogenesis^27^, and exhibit strong increases particularly during vertebrate evolution. Proteins involved in wnt signalling pathways are also pivotal in embryonic development and cell differentiation through cell fate, proliferation, and migration^28^. Palmitoyl transferases (EC:2.3.1.-), which perform reversible lipid modifications that are involved in neuronal development, protein trafficking, and synaptic plasticity^29^, belong to the transferases cluster that increases significantly throughout metazoans and more than doubles in most vertebrate species.

The evolution of the adaptive immune response is a metazoan hallmark with vast membrane proteome innovation and expansion. We show unique expansions in proteins that are well-known to perform crucial immunological roles in vertebrates^30,31^, such as the MHC 1 family which explodes in vertebrate species with one cluster having 22 to 46 proteins. The vertebrate IG and TOLL receptor cluster also shows fast developments, ranging from 18 to 65 proteins, while one cell surface receptor cluster ranges from 9 to 19 proteins. Other novel vertebrate proteins involved in immune response also show considerable numbers, such as CD molecules, proteins with the SHP2-interacting transmembrane adaptor protein family (PF15330), and receptors such as Ly49 (PF08391), PILRA-V-set domain (PF07686), and interferon. These developments are in coherence with earlier findings that suggest specific expansions of genomic components in the immune system occurred in vertebrates and can be reasoned by the fact that the adaptive immune system emerged at the origins of vertebrates in conjunction with coevolution with innate defences^2,32^.

Proteins involved in innate immunity, such as IG/TOLL and TIR (Toll-interleukin receptor) domain clusters are found throughout metazoans with distinct species specific expansions, for example reaching more than 250 proteins in *S*. *purpuratus* and nearly 140 in *L*. *anatina*. The rise of proteins with TIR and related domains allow specific functional responses and increase the variation that can be generated via innate immunity molecules to varying environmental challenges^33^. And in fact different innate immune system components can be seen to increase in different lineages, such as scavenger receptors and Lectin-like varied receptors. Studies show innate immune components include not only immune-related signalling, but are also involved in the co-adaptive evolution of stress^33^ and embryonic developments^34^. While the adaptive immune system that is based on lymphocytes expressing antigen receptors generated through somatic rearrangement of genes developed in vertebrates^2^ has been well studied, the diversity in innate immune responses in invertebrates seems to be more linage-specific and less understood^35^. Immune components such as pathogen recognition receptors and antimicrobial effectors provide some defence variation, however recent studies have investigated different manners to develop diversity. Other mechanisms to generate immune variation include processes that are not explored in this type of study, as they involve noncoding RNA sequences^36^ and also alternative splicing to produce many different isoforms, such as in Drosophila that uses the *Dscam* gene, which functions as an immune receptor, to generate more than 150,000 isoforms^37^.

Dynamic cytoskeleton organization with multicellular development also exhibit expansions within metazoans. The Growth-Arrest-Specific Protein 2 (Gas2) domain (PF02187) is conserved from the LHCA however it increases throughout metazoans, ranging from 2 to 15 proteins. This domain is common in plakin family members, which in turn form critical junctions between cell junctions and the cytoskeleton^38^. Previous investigation of the Gas2 protein suggests a conserved biological function among vertebrates that includes playing a role in cell division and that its function is mediated by bundling microtubules^39^. However, the function of this protein family in unicellular relatives has not been investigated. Proteins with the conserved SUN domain (PF07738), which double to four proteins in vertebrates and 6 proteins in mammals, are proposed to serve as mechanical adaptors and nuclear envelope receptors that connect inner and outer nuclear membranes, and also linking the cytoskeleton and nucleoskeleton^40^.

One area that invites more investigation is the potential effects of the environment and lifestyle of a species that can possibly be seen in both the expansion of certain protein families but also in reductions in the size of a genome and subsequently the subset of membrane proteins. The ecdysozoans *D*. *melanogaster* and *A*. *mellifera* both show decreased genome sizes (see Table 1: Membrane Proteome Totals) and marked losses or reductions in the number of proteins within a cluster. Wyder *et al*. studied the quantification of orthologue losses in insects and vertebrates and concluded that the rate of losses correlates with the species’ rates of molecular evolution and radiation times, indicating that the observed gene losses in these ecdysozoans are explained by their higher evolutionary rate^41^. While the reasons behind the gene loss in these ecdysozoans and other species is not fully understood, Abalat and Cañestro review the evolution of gene loss and discuss adaptive gene loss and ‘environment-dependent conditional dispensability’ in environments where the function of a gene is not needed^42^. The scope of genomes presented here, ranging from opisthokonta and including 17 invertebrates, with 11 protostomes and 7 deuterostomes, present gene losses and expansions with as complete a context possible for the data presently available.

Several interesting sets of clusters include the vertebrate GPCR Taste receptors and the invertebrate Chemosensory receptors, which both seem to detect virtually the same classes of chemicals. However the receptors do not appear to be evolutionarily conserved, and in fact the invertebrate chemosensory receptors may form ligand-activated ion channels instead of classical GPCRs^43^. Taste allows not only detection of nutrients but also warns of toxic substances and further sensing and adapting to highly variable chemical environments^44^. The vertebrate GPCR Taste receptors show pronounced lineage specific expansions while three of the four ecdysozoans show large expansions in this cluster– ranging from 49 to 61 proteins, which could be attributed to adaptive sensory evolution exploiting various ecological niches.

Overall, we can infer that the complexity inherent in the membrane proteome was already present early in the last holozoan common ancestor and that the membrane bound genome content underwent multiple large-scale expansions at the origins of metazoans, in species specific inflations, as well as in vertebrates. Further, later reductions in genome size and gene loss are evident and the causes for these phenomena are still being studied. This investigation highlights the role of membrane proteomes in the evolution of metazoans and several of its key morphological features.

## Methods

### Proteome retrieval

The whole proteomes for the twenty-four genomes were retrieved from publicly available databases: *Homo sapiens (GRCh38*.*p3)*, *Mus musculus*, *Ciona intestinalis*, *Danio Rerio*, *Gallus gallus*, *and Xenopus tropicalis* were downloaded from Ensembl Release 27 and *Apis mellifera* and *Crassostrea gigas* from Release 35 (http://www.ensembl.org/index.html); *Amphimedon queenslandica*, *Drosophila melanogaster*, *Caenorhabditis elegans*, *Nematostella vectensis*, *Schistosoma mansoni*, *Strongylocentrotus purpuratus*, *Trichoplax adhaerens* were retrieved from Ensembl Metazoa Release 27 (http://metazoa.ensembl.org/index.html); *Capsaspora owczarzaki* was downloaded from the Broad Institute of Harvard and MIT (www.broadinstitute.org); *Mnemiopsis leidyi* was retrieved from the National Human Genome Research Institute (http://research.nhgri.nih.gov/mnemiopsis/); *Daphnia pulex*, *Capitella teleta*, *Lottia gigantea* filtered models and *Monosiga brevicollis* were downloaded from the JGI Genome portal (http://genome.jgi-psf.org/); *Lingula anatina* v1.0 was obtained from the OIST Marine Genomics website (http://marinegenomics.oist.jp); *Acyrthosiphon pisum* was downloaded from the BioInformatics Platform for Agroecosystem Arthropods (http://bipaa.genouest.org/is/); and *Intoshia linei* was retrieved from the National Center for Biotechnology Information (https://www.ncbi.nlm.nih.gov/). As genome assemblies can be difficult to assess, even the most common metric used, N50, may not accurately reflect the quality of the assembly. Considerations such as coverage, size of the genome, and length of reads can affect the quality of the assembly and subsequently miss genes that may actually be present in the genome^45^. Additionally, gene prediction pipelines can also be problematic with issues such as identifying and masking repeat sequences, correctly aligning ESTs and RNA-seq data to the genome assembly, and automatically annotating the predicted genes resulting in missing or incorrectly annotated genes^46^. To that end, the downloaded species proteomes were chosen with consideration to the best available resources, i.e., using the Genome Reference Consortium as well as other consortiums with high quality assemblies and gene prediction pipelines. To prepare the sequences for analysis, each proteome was assessed and if a gene produced multiple protein isoforms, the longest sequence for each gene was used.

### Prediction of membrane proteomes

The membrane proteome of an organism was defined as all proteins that contained alpha-helical transmembrane spanning regions. As transmembrane protein prediction methods can have difficulty differentiating between signal peptides and transmembrane segments, signal peptides need to be assessed and excised from the protein sequences. A local instalment of SignalP 4.1 was used with default settings to detect signal peptides and retrieve the mature sequences as appropriate^47^. Previous studies have shown that using multiple transmembrane protein prediction methods can give more accurate prediction results^48^ and thus the consensus method TOPCONS-single, which is suitable to use for large proteome datasets, was used through the web server (http://single.topcons.net/)^49^. TOPCONS-single is a consensus prediction method that incorporates multiple methods and uses a hidden-Markov model to estimate the consensus topology from the methods for a predicted transmembrane protein. As recommended by the authors, the four methods used were: SCAMPI-single^50^; S-TMHMM^51^; HMMTOP^52^; and MEMSAT^53^. To ensure that all predicted membrane proteins were valid proteins with acceptable transcriptional support and recognized protein-coding annotation, the Reference Sequence identifier was used to assess the predicted dataset.

### Annotation of Pfam families

All proteins included in the membrane proteome dataset were searched against the Pfam^54^ database (v28) using an instalment on the UPPMAX high-performance computing service. The script *pfam_scan*.*pl* was used with default settings to obtain the associated Pfam families and domains for each protein.

### Two-level clustering of membrane proteins

The membrane proteins of all species were grouped through two levels of clustering: the first level was based on BLAST comparisons and the second level on Pfam family composition. The first level of clustering used the BLAST+ package^55^ to create a local database and then perform all-versus-all pairwise alignments. An e-value threshold of 0.01 and the *blastp* method was applied along with default settings. A homology network was constructed from the BLAST results and clusters of homologous proteins were identified using the Markov Cluster (MCL) algorithm^56^, which has been successfully applied to detect protein families on a large scale from BLAST searches for several large projects, such as Ensembl Compara^57^. The recommended MCL workflow for processing BLAST searches suggested using the *mcxload* program of the MCL package to initially write a native network file and a dictionary file. The BLAST results were first parsed into a tab delimited file (-*abc*) and then processed as an undirected network (–*stream-mirror*) with edges weighted by the negative logarithm of the e-value (–*stream-neg-log*1*0*) and a maximum weight of 200 (–*stream-tf* ‘*ceil(200*)’). The clusters were then calculated using the *mcl* program with default settings and an inflation value of 1.6 (−*I 1*.6).

The second level of clustering grouped the first level clusters based on Pfam families. Only first level clusters that had proteins with a Pfam and/or a function associated with them were included in the second level clustering. Additionally, only Pfam models of the “family” type were considered to avoid clustering on repeats or promiscuous domains. Two clusters that contained proteins of the same Pfam family were connected in the network with an edge weighted by the fraction (0.0–1.0) of proteins that contained that Pfam family. The edge weight was based on the cluster with the highest fraction and a weight threshold of 0.5 was applied. The network was parsed into an MCL native network file using *mcxload* with default settings and then clustered using *mcl* with an inflation value of 1.8 (−*I* 1.8) and weight threshold 0.5 (−*tf* ‘*gq*(0.5)’). While this method of detecting distant homologues has been used previously, Martín-Durán *et al*. recently published a study on hidden orthologues in flatworms, which are sequences with no apparent homology to sequenced animal lineages and subsequently mistaken for new genes, however in actuality they are rapidly evolving orthologues or undetected paralogues^58^. This would possibly have an effect in this type of study, perhaps revealing some of the uncharacterized proteins to be members of known families. As more species genomes are sequences, this information can be used along with new methods such as Leapfrog presented in this paper to detect missed homologues.

### Classification of proteins and clusters

The final classifications of the second level clusters were based on several elements: the characterizations of the human membrane proteome and subsequent cluster representations; and additional Pfam family descriptions. Initially, the human membrane proteome was annotated by cross-referencing UniProt (http://www.uniprot.org/) and extracting the associated information for each protein: Enzyme Commission number (EC), Transporter Classification Database identification (TCDB), and the Function Summary assigned to each protein (if possible). Additionally, the ‘target and family’ and ‘ligand’ lists from IUPHAR/Guide to Pharmacology (http://www.guidetopharmacology.org/download.jsp) were downloaded and assessed as well. Proteins that had an associated EC number were labelled *enzymes* and those that had an associated TCDB identifier were labelled *transporters*. All other proteins were manually curated using the downloaded IUPHAR lists, Function Summary, and Pfam family descriptions and grouped into either *receptors* or *other functional classification* and then further characterized with several discrete descriptions. Those proteins with an associated conserved Pfam family that had a domain of unknown function (DUF) were duly noted.

The human protein characterizations for each first level clustering was assessed and a consensus first level cluster description was obtained. The second level clusters were then manually controlled for conflicting classifications of the underlying proteins and an appropriate characterization was determined for each second level cluster. Some clusters were deemed ambiguous as consensus classification failed. There are some second level clusters that do not contain any characterized human proteins however there was an associated Pfam family identified and used to characterize the cluster.

To further validate the methodology and clustering, a subset of the data (~23%) was tested with OrthoFinder^59^, which identifies distant homology relationships in proteins. The largest 10 orthogroups from the OrthoFinder subset results were checked against our dataset, with all except one group having greater than 92% consistency between the two sets. The one outlier was a group containing several clusters that we have identified as having Varied Pfam; i.e., there was neither a consistent human protein functional description nor Pfam annotation that could conclusively define these clusters.

All analyses and classifications were performed using local Python and Perl scripts and SQL databases (sqlite3). For the figures, the data was standardized using the R data.Normalization function from the clusterSim package with the n1 parameter. Adobe Illustrator was used for additional information in the figures.

## Electronic supplementary material

Supplemental Data S1

Supplemental Table S2
