# Supplementary material for: Highly diversified expansions shaped the evolution of membrane bound proteins in metazoans
Source: Sci Rep. 2017 Sep 28;7:12387. doi: 10.1038/s41598-017-11543-z (PMC5620054; doi:10.1038/s41598-017-11543-z)
Supplement: Supplementary file 2 — Supplemental Table S2 [file 41598_2017_11543_MOESM2_ESM.pdf]

Highly diversified expansions shaped the evolution of membrane bound proteins in metazoans

Misty M Attwood, Arunkumar Krishnan, Markus Sällman Almén, and Helgi B Schiöth*

Supplemental Table S2. Species specific clusters, i.e. only one species identified in each cluster, of Conserved DUF clusters (top of tables) and Uncharacterized sequences (bottom of tables). The left column identifies the number of proteins in a species specific cluster, e.g. in Uncharacterized clusters that contain 2 members, *M. musculus* has 12 proteins identified, which is 6 different clusters with two members each. The numbers of proteins are shown with the number of clusters in parenthesis.

Deuterostomia	H. sapiens	M. musculus	G. gallus	X. tropicalis	D. rerio	C. intestinalis
Conserved DUF						
**1**	33	3	2	2	-	-
**2**	2	-	2	-	-	-
Uncharacterized						
**1**	-	85	90	18	98	522
**2**	-	12 (6)	-	2	32 (16)	54 (27)
**3**	-	12(4)	3	-	24 (8)	18 (6)
**4**	-	-	-	-	-	20 (5)
**6**	-	-	-	-	6	-
**8**	-	8	-	-	8	-
**12**	-	12	-	-	-	-
**13**	-	-	-	-	26 (2)	-
**16**	-	-	-	-	16	-
**Totals**	35	132	97	22	210	644

Protostomia	D. melanogaster	A. pisum	D. pulex	C. elegans	C. teleta	L. anatina	C. gigas	L. gigantea
**Conserved DUF**								
**1**	5	2	1	2	6	-	-	1
**2**	-	-	-	6 (3)	4 (2)	2	-	-
**3**	6	-	3	3	-	-	-	-
**4**	4	-	-	4	-	-	-	-
**6**	6	-	-	6	-	-	-	-
**7**	-	-	7	7	-	-	-	-
**8**	-	-	-	8	-	-	-	-
**9**	-	-	-	9	-	-	-	-
**10**	-	10	-	20 (2)	-	-	-	-
**11**	-	-	-	11	-	-	-	-
**12**	-	-	-	12	-	-	-	-
**Uncharacterized**								
**1**	242	626	684	920	450	309	377	310
**2**	38 (19)	100 (50)	152 (76)	188 (94)	154 (77)	382 (191)	134 (67)	80 (40)
**3**	12 (4)	57 (19)	78 (26)	87 (29)	60 (20)	144 (48)	75 (25)	60 (20)
**4**	16 (4)	44 (11)	76 (19)	36 (9)	52 (13)	100 (25)	44 (11)	16 (4)
**5**	15 (3)	45 (9)	50 (10)	35 (7)	30 (6)	65 (13)	25 (5)	35 (7)
**6**	-	-	30 (5)	24 (4)	48 (8)	30 (5)	18 (3)	18 (3)
**7**	7	7	35 (5)	42 (6)	28 (4)	35 (5)	7	-
**8**	-	16 (2)	16 (2)	16 (2)	-	16 (2)	8	-
**9**	-	9	18 (2)	9	9	18 (2)	-	27 (3)
**10**	-	-	10	10	-	-	-	20 (2)
**11**	-	11	-	-	-	22 (2)	-	-
**12**	-	-	12	12	-	-	-	-
**13**	-	13	-	-	-	-	-	-
**14**	-	14	14	-	14	-	-	-
**17**	-	-	-	17	-	-	-	-
**18**	-	-	-	-	18	18	-	-
**19**	-	-	-	38 (2)	-	-	-	-
**20**	-	-	-	20	-	-	-	-
**22**	-	-	-	22	-	-	-	-
**25**	25	-	-	25	-	-	-	-
**40**	-	-	-	-	40	-	-	-
**72**	-	-	-	72	-	-	-	-
**Totals**	388	966	1186	1661	913	1141	688	567

	I. linei	S. mansoni	N. vectensis	T. adhaerens	M. leidyi	A. queenslandica	M. brevicollis	C. owczarzaki
Conserved DUF								
**1**	-	1	3	4	1	4	7	9
**2**	-	-	3	2	-	2	-	2
**13**	-	-	13	-	-	-	-	-
Uncharacterized								
**1**	181	392	190	128	454	405	367	285
**2**	34 (17)	38 (19)	50 (25)	42 (21)	90 (45)	104 (52)	58 (29)	28 (14)
**3**	-	12 (4)	24 (8)	21 (7)	27 (9)	60 (20)	12 (4)	15 (5)
**4**	8 (2)	4	28 (7)	8 (2)	44 (11)	36 (9)	16 (4)	16 (4)
**5**	-	10 (2)	10 (2)	-	5	25 (5)	15 (3)	5
**6**	6	6	-	6	6	18 (3)	-	18 (3)
**7**	-	-	7	-	7	7	-	-
**8**	8	-	-	8	16 (2)	8	-	-
**9**	-	-	-	-	-	18 (2)	-	9
**11**	-	-	-	-	11	11	-	-
**12**	12	-	-	-	-	-	-	-
**13**	-	-	-	-	13	-	-	-
**14**	-	-	-	-	-	-	-	14
**15**	-	-	-	-	-	-	-	15
**21**	-	-	-	-	-	42 (2)	-	-
**22**	-	-	-	22	-	22	-	-
**29**	-	-	29	-	-	-	-	-
**Totals**	249	463	357	241	684	762	475	417
